# The complete chloroplast genome of *Waldheimia glabra*

**DOI:** 10.1080/23802359.2021.1944372

**Published:** 2021-07-06

**Authors:** Wang Hai, Weiyu Wang, Li Tong, Nie Shao-Hu, Wei Li, Yi-Zeng Lu, Bo-Qiang Tong, Zhao Hui-En

**Affiliations:** a Beijing Key Laboratory of Ornamental Plants Germplasm Innovation & Molecular Breeding; National Engineering Research Center for Floriculture, Key Laboratory of Genetics and Breeding in Forest Trees and Ornamental Plants of Ministry of Education, College of Landscape Architecture, Beijing Forestry University, Beijing, China; b International Education College, Nanjing Forestry University, Nanjing, China; c College of Landscape Architecture and Forestry, Qingdao Agricultural University, Qingdao, China; d Shandong Provincial Center of Forest Tree Germplasm Resources, Jinan, China

**Keywords:** Asteraceae, chloroplast genome, phylogenetic relationships, *Waldheimia glabra*

## Abstract

*Waldheimia glabra* (Decne.) Rgl. 1879 (family Asteraceae) is a perennial herb with high economic and medicinal values. In this study, we sequenced the complete chloroplast (cp) genome of *W. glabra* by high-throughput Illumina sequencing. The size of the *W. glabra* cp genome is 151,499 bp, with overall GC content of 37.3%. It contains a large single copy and a small single copy region of 83,078 bp and 18,457 bp, respectively, separated by a pair of inverted repeats regions of 24,982 bp. We also discovered 131 genes, including 86 protein-coding genes, 37 transfer RNA genes, and 8 ribosomal RNA genes in the genome. The maximum-likelihood phylogenetic tree demonstrated that *W. glabra* is closely related to *Leucanthemella linearis*.

*Waldheimia glabra*, a perennial herb (family Asteraceae), is mainly distributed in India, Nepal, Pakistan, Afghanistan, and China, growing on the stony slopes of the Himalayas at 4000 − 5400 m altitude. The plant is medicinally important and is often used to treat common infections, skin diseases, arthritis, and rheumatism in traditional medicine. It is also used as vermifuge, antiseptic paste, and incense (Bhatnagar et al. 2017). Although it has high economic and medicinal values, reports on the floristic study of this high-altitude plant species are limited. Consequently, germplasm resources are decreasing, and breeding and applications are restricted.

The study of the genetic variability of *W. glabra* to formulate appropriate conservation strategies and developing resistant fine cultivars requires immediate attention. The objectives of our study were to sequence and assemble the complete chloroplast genome (cp) of *W. glabra* using next generation sequencing (Du et al. 2015). We also analyzed its phylogenetic relationship with other members of the Asteraceae family for better understanding of their interspecific relationship. Our study facilitates the protection of *W. glabra* germplasm resources.

The plant material of *W. glabra* was collected from the Himalayan region of Burang County, Ngari Prefecture, Tibet, China (N 30°12′29.3″, E 81°37′22.3″) and the specimen was deposited at Museum of Beijing Forestry University, Beijing, China (Huien Zhao, zhaohuien@bjfu.edu.cn, 20180829BJFU010). Total genomic DNA was extracted from fresh young leaves (Li et al. 2013; Xu et al. 2019) and conserved in Beijing Forestry University. The extracted DNA was subjected to paired-end libraries construction and sequencing on an Illumina Novaseq platform. The results were stored in the FASTQ file format.

Approximately 4.25 Gb of clean data were generated after filtering. The GetOrganelle (Jin et al. 2018) and SOAPdenovo software (Luo et al. 2012) assessed and assembled the paired-end reads. We used OGDRAW program to draw circular chloroplast genome map (Marc et al. 2013). The cp genome sequence of *W. glabra* has been submitted to GenBank (accession number: MW628520).

The cp DNA of *W. glabra* is 151,499 bp long, with an average sequencing depth of 467×. It contains a large single-copy (LSC) region of 83,078 bp and a small single-copy (SSC) region of 18,457 bp, which is separated by a pair of inverted repeats (IR) regions (24,982 bp). The overall GC content of the cp genome is 37.3%. We annotated a total of 131 functional genes, including 86 protein-coding genes, 8 ribosomal RNA (rRNA) genes, and 37 transfer RNA (tRNA) genes. Among them, 15 genes contain only one intron and 2 genes contain two introns. In addition, one ribosomal-protein gene has trans-splicing. Gene duplication was found in the IR regions, including one protein-coding gene, four tRNA genes, and four rRNA genes. Our result is consistent with those obtained from other Asteraceae species (Zhang et al. 2016).

Since reports on the floristic study of this high-altitude plant species are limited, its interspecific relationship with other members of the family Asteraceae is still unclear. A phylogenetic analysis compared the cp genome of *W. glabra* with those from 21 Asteraceae species, including *Chrysanthemum*, *Artimisia*, *Crossostephium*, *Opisthopappus*, and other genera. *Arabidopsis thaliana* was used as an outgroup for the study. The maximum-likelihood phylogenetic tree shows that *W*. *glabra* is most closely related to *Leucanthemella linearis* with high bootstrap values. It clusters with *Stilpnolepis centiflora*, *Chrysahthemum*, and *Artimisia. Chrysanthemum* and *Artimisia* are grouped together (Figure 1). Our result is consistent with previous studies on the phylogenetic relationship of Asteraceae species (Panero and Crozier 2016; Wang et al. 2020). Our research lays the foundation for the study of genetic diversity and phylogeny of *W*. *glabra*.

**Figure 1.**
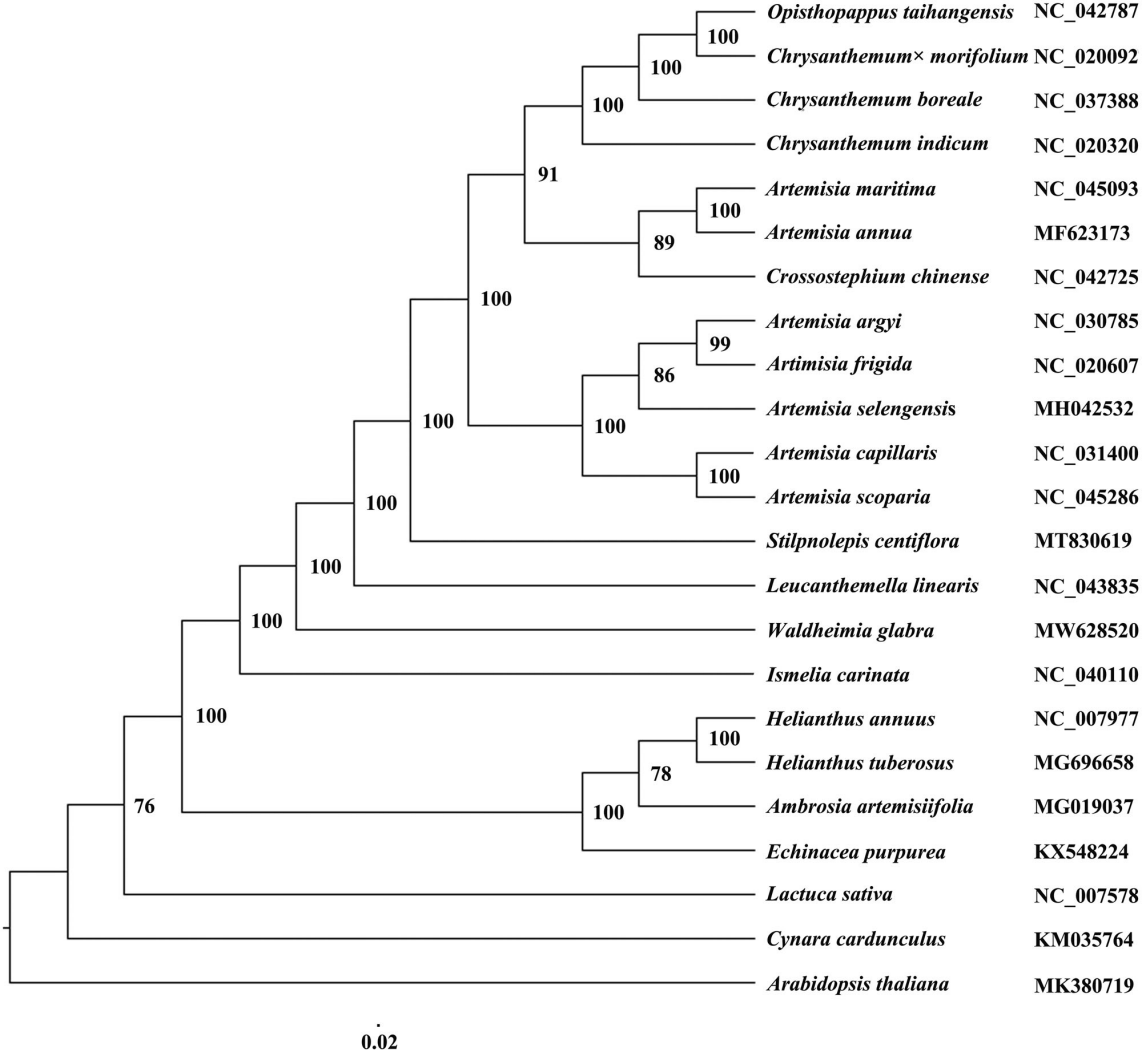
Phylogenetic relationships of *W. glabra* and the other 21 Asteraceae species based on the cp genome sequences and Arabidopsis thaliana was used as an outgroup.

## Data Availability

The genome sequence data that support the findings of this study are openly available in the GenBank of NCBI at (https://www.ncbi.nlm.nih.gov) under the accession no. MW628520 (https://www.ncbi.nlm.nih.gov/nuccore/MW628520.1/). The associated BioProject, SRA and Bio-Sample number are PRJNA702561 (https://www.ncbi.nlm.nih.gov/bioproject/PRJNA702561), SRR14664915 and SAMN19356817 respectively.

## References

[CIT0001] Bhatnagar M, Avasthi AS, Singh S, Ghosal S. 2017. Evaluation of anti-leishmanial and antibacterial activity of *Waldheimia tomentosa* (Asteraceae), and chemical profiling of the most bioactive fraction. Trop J Pharm Res. 16(9):2169–2178.

[CIT0002] Du FK, Lang T, Lu S, Wang Y, Li J, Yin K. 2015. An improved method for chloroplast genome sequencing in non-model forest tree species. Tree Genet Genomes. 11(6):114.

[CIT0003] Jin J, Yu WB, Yang JB, Song Y, Li DZ, Yi T. 2018. GetOrganelle: a simple and fast pipeline for de novo assembly of a complete circular chloroplast genome using genome skimming data. bioRxiv. 256479.

[CIT0004] Li J, Wang S, Yu J, Zhou S. 2013. An improved method for extracting plant DNA. Chin Bull Bot. 48(1):78–78.

[CIT0005] Luo R, Liu B, Xie Y, Li Z, Huang W, Yuan J, He G, Chen Y, Pan Q, Liu Y, et al. 2012. SOAPdenovo2: an empirically improved memory-efficient short-read *de novo* assembler. Gigascience. 1(1):18–18. 23587118 10.1186/2047-217X-1-18 PMC3626529

[CIT0006] Marc L, Oliver D, Sabine K, Ralph B. 2013. OrganellarGenomeDRAW – a suite of tools for generating physical maps of plastid and mitochondrial genomes and visualizing expression data sets. Nucleic Acids Res. 41(W1):W575–W581. 23609545 10.1093/nar/gkt289 PMC3692101

[CIT0007] Panero JL, Crozier BS. 2016. Macroevolutionary dynamics in the early diversification of Asteraceae. Mol Phylogenet Evol. 99:116–132. 26979262 10.1016/j.ympev.2016.03.007

[CIT0008] Wang J, Qian Q, He R, Zhang H, Wang J, Hu Y, Wang L, Li Y. 2020. The complete chloroplast genome of *Saussurea medusa* (Asteraceae), a rare subnival plant in Qinghai-Tibetan Plateau. Mitochondrial DNA Part B. 5(3):3563–3564. 10.1080/23802359.2020.1829130 PMC7594724 33367018

[CIT0009] Xu ZZ, Zhang CP, Jiang XQ, Guo X, Li WQ, Liu QH, Wang KL, Li W. 2019. The complete chloroplast genome of *Styrax japonicus*. Mitochondrial DNA Part B. 5(1):81–82. 33366432 10.1080/23802359.2019.1676175 PMC7720720

[CIT0010] Zhang JM, Huang B, Chen XL. 2016. The complete chloroplast genomes of *Asteraceae* species. Res Rev J Bot Sci. 5:24–28.

